# Impaired cardiopulmonary functions in prepubertal patients with Kawasaki disease

**DOI:** 10.1016/j.clinsp.2025.100773

**Published:** 2025-09-11

**Authors:** Yen-Sen Lu, I-Ching Huang, Yen-Hsien Wu, Sheng-Hui Tuan, Yi-Ching Liu, Yi-Cheng Wang, Shih-Hsing Lo, Jong-Hau Hsu, Ko-Long Lin

**Affiliations:** a Department of Physical Medicine and Rehabilitation, Kaohsiung Medical University Hospital, Kaohsiung Medical University, Kaohsiung, Taiwan; b Department of Physical Medicine and Rehabilitation, Kaohsiung Municipal Ta-Tung Hospital, Kaohsiung, Taiwan; c Department of Pediatrics, Kaohsiung Medical University Hospital, Kaohsiung Medical University, Kaohsiung, Taiwan; d Institute of Allied Health Sciences, College of Medicine, National Cheng Kung University, Taiwan; e Department of Rehabilitation Medicine, Cishan Hospital, Ministry of Health and Welfare, Taiwan; f Department of Physical Medicine and Rehabilitation, Faculty of Medicine, College of Medicine, Kaohsiung Medical University, Kaohsiung, Taiwan; g School of Medicine, College of Medicine, Kaohsiung Medical University, Kaohsiung, Taiwan

**Keywords:** Cardiopulmonary exercise testing, Exercise capacity, Kawasaki Disease, Prepubertal Period

## Abstract

**Objective:**

This study investigated the cardiopulmonary function of prepubertal patients with Kawasaki Disease (KD) and compared it with that of healthy controls.

**Study design:**

Retrospective cohort study

**Methods:**

Data were collected from two medical centers in Taiwan from January 2014 to December 2023. Patients aged 8 to 12 years with a KD history were recruited. All patients and controls (age-, sex-, and body mass index -matched) underwent Cardiopulmonary Exercise Test (CPET) and pulmonary function test.

**Results:**

A total of 117 KD patients (mean age 9.64 ± 1.66 years; 68 males (58.1 %); (Body Mass Index [BMI] 19.21 ± 4.20 kg/m^2^) and 121 controls (mean age 9.72 ± 1.66 years; 71 males (58.7 %); BMI 18.47 ± 3.30 kg/m^2^) were analyzed. The average time from illness onset to enrollment was 7.94 ± 2.84 years. No significant differences in demographic characteristics or pulmonary function were observed. However, the KD group exhibited lower aerobic capacity (24.32 ± 5.02 vs. 26.08 ± 4.89 mL/min/kg, *p* < 0.001) and peak exercise tolerance (35.43 ± 7.31 vs. 38.48 ± 6.24 mL/min/kg, *p* < 0.001) compared to controls. Furthermore, the KD group had lower cardiopulmonary function than the control group among males, whereas the difference in females did not reach statistical significance.

**Conclusions:**

Prepubertal individuals with a KD history demonstrated lower aerobic capacity and peak exercise tolerance than healthy peers, particularly among males. Despite this, they can safely engage in moderate-to-vigorous physical activities, which are essential for maintaining cardiopulmonary health in later life. Encouraging regular exercise among these patients is crucial.

## Highlights

• Prepubertal children with Kawasaki disease have lower aerobic capacity and exercise tolerance.
• Males with Kawasaki disease are more severely affected in cardiopulmonary function than females.
• Despite reduced capacity, KD children can safely engage in vigorous physical activities.
• Regular exercise is crucial for improving long-term cardiopulmonary health in KD patients.

## Introduction

Kawasaki Disease (KD), also known as mucocutaneous lymph node syndrome, is an acute, self-limited febrile illness of unknown cause that predominantly affects children under 5-years of age. The clinical features of KD reflect widespread inflammation of primarily medium-sized muscular arteries, particularly the Coronary Arteries (CAs), leading to Coronary Artery Abnormalities (CAAs), which makes KD currently the most common cause of acquired heart disease in children in developed countries.^1^ Severe complications, such as thrombosis, stenosis, and myocardial infarction, may also occur secondary to the coronary artery aneurysm or ectasia. Therefore, early detection and timely intervention of CAAs in KD are crucial to prevent these complications. Intervention typically involves intravenous immunoglobulin and aspirin therapy, which can help reduce inflammation and prevent the CA aneurysms. However, although most children with KD respond well to standard treatment, 5 % of treated children may still develop CA aneurysms, and 1 % will develop giant aneurysms with severe cardiac sequela.^2^, ^3^, ^4^ Hence, long-term follow-up of affected children and adults is crucial.

Notably, previous studies comparing cardiopulmonary function in children between KD and normal children have yielded inconsistent conclusions, despite the risk of developing CAAs. Both Tuan et al. and Gravel et al. revealed that although children with KD had lower rates of myocardial perfusion during exercise, their cardiopulmonary function and exercise load capacities were comparable to those of healthy children.^5^^,^^6^ Other recent studies have found that adolescents with a KD history had significantly lower aerobic metabolism and peak exercise load capacities than controls.^7^^,^^8^ Furthermore, studies exploring KD have mainly focused on children over the age 12-years, and no studies have yet been conducted regarding the cardiopulmonary functions of prepubertal individuals who had KD in childhood. Therefore, the purpose of this study was to investigate the cardiopulmonary function of prepubertal patients with KD.

## Material and methods

### Patient selection and data collection

This retrospective cohort study analyzed data collected at two medical centers in Taiwan from January 2014 to December 2023. Children aged between 8 and 12 years who were referred from the pediatric cardiology outpatient clinic for KD follow-up were recruited. The exclusion criteria included individuals with congenital heart diseases (e.g., ventricular septal defect and patent ductus arteriosus), with current or history of significant arrhythmia, moderate to severe valvular heart disease, coronary artery diseases not caused by KD, and known concurrent pulmonary disease (e.g., asthma). Patients with missing data were also excluded.

For comparison, the control group comprised age-, sex-, and Body Mass Index (BMI)-matched children who were referred from the pediatric cardiology outpatient clinic of the same medical centers in Taiwan during the same period for chest pain or dyspnea on exertion, but were diagnosed as healthy individuals following a series of examinations, including physical examinations by pediatricians, echocardiography, and 12-lead electrocardiography. All patients underwent body weight and height measurements, followed by CPET and pulmonary function tests. Informed consent was obtained from the parents of all patients before the examinations.

This study was approved by the Institutional Review Boards of Kaohsiung Veterans General Hospital (VGHKS 17-CT11–11) and Kaohsiung Medical University Hospital (KMUHIRB-E(I)-20,240,166).

### CPET

To evaluate the exercise capacity of the participants, a graded symptom-limited exercise testing system was employed, which comprised a treadmill, flow module, gas analyzer, and electrocardiographic monitor (Metamax 3B, Cortex Biophysik GmbH Co., Germany), and both medical centers used the same equipment for the evaluation. Before beginning the examination, the purpose of the test was thoroughly explained to the participants and his or her parents. All participants completed tests according to the Bruce/Ramp protocol suggested by the American College of Sports Medicine (ACSM).^9^ The test was terminated when the patient encountered subjectively unbearable symptoms, could not continue, or had attained maximum exercise capacity, as indicated by the ACSM. The Oxygen consumption (VO_2_) and Carbon Dioxide production (VCO_2_) were measured by the breath-by-breath method during the testing. Furthermore, blood pressure, heart rate, minute Ventilation (V_E_), and Respiratory Exchange Ratio (RER) were measured throughout the testing. The RER value was calculated as follows: VCO_2_/VO_2_. The VO_2_ at Anaerobic Threshold (AT VO_2_) and the peak exercise (peak VO_2_) were also determined. AT was determined by the V_E_/VO_2_ and V_E_/VCO_2_ methods.^10^ Peak VO_2_ was defined as the maximum oxygen uptake measured during peak exercise. Peak exercise was determined when two of the following three conditions were met: 1) RER > 1.1, 2) Heart rate within 5 % of the age-predicted maximum, and 3) The participant was exhausted and refused to continue the test despite strong verbal encouragement.^11^^,^^12^ All tests were performed smoothly under the supervision of a well-trained physiatrist (K.L.L) who has >20 years of experience in CPET.

### Pulmonary function test

All subjects underwent pulmonary function tests by spirometry at rest. Forced Vital Capacity (FVC), Forced Expiratory Volume in 1 s (FEV_1_), as well as Maximal Voluntary Ventilation (MVV) were assessed. The predicted value of each spirometry measure was calculated based on the spirometric reference equations for healthy children in Taiwan.^13^

### Statistical analyses

All statistical analyses were performed with SPSS for Windows version 20.0 (Released 2011; IBM Corp, Armonk, NY). Continuous data are expressed as means ± standard deviations, and categorical variables are presented as absolute numbers or percentages. Normality and homoscedasticity were examined before each analysis. Baseline characteristics and cardiopulmonary exercise parameters between the KD and control groups were compared by the independent *t*-test for continuous variables if they conform to normal distribution; if they did not conform to normal distribution, they were compared by the Mann-Whitney *U* test. Furthermore, the chi-square test was used to compare categorical variables. A pre-specified two-sided alpha of 0.05 and 95 % Confidence Intervals were used to determine statistical significance.

## Results

**Fig. 1:**
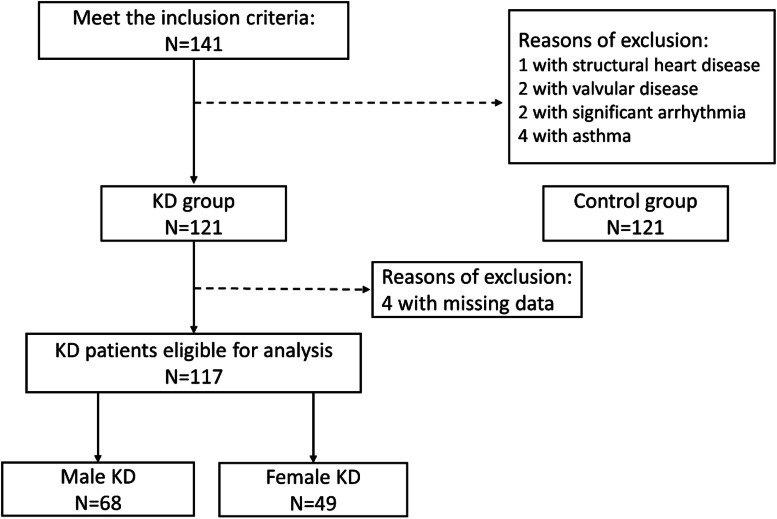
Flowchart illustrating the inclusion process of patient selection.

In total, 130 patients met the inclusion criteria. Among them, two patients with significant cardiac structural problems, one patient with valvular heart disease, two patients who presented with significant arrhythmia, four patients with asthma, and four patients with missing data were excluded. Fig. 1 presents the patient selection process. Eventually, 117 patients with KD were recruited for the final analysis. Table 1 shows the demographic characteristics of the KD and control groups. The average age of all children with KD was 9.64 ± 1.66 years, with 58.1 % being males. Additionally, the average time from illness onset to enrollment was 7.94 ± 2.84 years; the average age of control children was 9.72 ± 1.66 years, with 58.7 % being males. No statistically significant differences in sex, age, weight, height, BMI, body fat, systolic and diastolic blood pressures, or resting heart rate were observed between the KD and control groups. Table 2 shows the results of the pulmonary function tests and CPET of the KD and control groups. No statistical difference in the results of the pulmonary function tests was found between the two groups. Regarding performance in CPET, all subjects achieved a maximum exercise level indicated by a peak RER of at least 1.1, and the results showed lower AT VO_2_ (24.32 ± 5.02 mL/min/kg in KD; 26.08 ± 4.89 mL/min/kg in Control, *p* < 0.001) and peak VO_2_ (35.43 ± 7.31 mL/min/kg in KD; 38.48 ± 6.24 mL/min/kg in Control, *p* < 0.001) in the KD group than in the control group.

**Table 1:** Demographic characteristics of KD and control groups.

	KD	Control	p-value
N	117	121	
**Age, years**	9.64 (±1.66)	9.72 (±1.66)	0.718
**Sex, n** ( %)			0.930
Male	68 (58.1 %)	71 (58.7 %)	
Female	49 (41.9 %)	50 (41.3 %)	
**Height, cm**	138.14 (±12.88)	137.70 (±11.32)	0.779
**Body weight, kg**	37.71 (±13.30)	35.44 (±10.07)	0.139
**BMI, kg/m^2^**	19.21 (±4.20)	18.47 (±3.30)	0.131
**Resting SBP, mmHg**	110.04 (±13.44)	109.48 (±11.66)	0.730
**Resting DBP, mmHg**	65.81 (±9.30)	64.87 (±9.47)	0.439
**Resting HR, bpm**	88.67 (±11.59)	88.16 (±11.50)	0.734

Data are the mean ± standard deviation or numbers ( %).

BMI, Body Mass Index; DBP, Diastolic Blood Pressure; HR, Heart Rate; KD, Kawasaki Disease; SBP, Systolic Blood Pressure.

**Table 2:** Performance of pulmonary function test and exercise test of KDs and control groups.

	KDs	Control	p-value
Performance of pulmonary function test			
FVC, L	2.05 (±0.63)	1.95 (±0.48)	0.235
FVCP, %	101.61 (±21.12)	99.10 (±20.37)	0.390
FEV1, L	1.79 (±0.53)	1.72 (±0.46)	0.297
FEV1P, %	101.56 (±24.85)	98.19 (±24.72)	0.337
FEV1/FVC, %	87.33 (±10.93)	87.55 (±9.01)	0.870
MVV, L/min	51.14 (±19.48)	49.37 (±15.41)	0.486

Performance of exercise test			
AT VO_2_, mL/kg/min	24.32 (±5.02)	26.08 (±4.89)	<0.001
AT HR, bpm	142.93 (±13.26)	145.27 (±9.72)	0.123
Peak VO_2_, mL/kg/min	35.43 (±7.31)	38.48 (±6.24)	<0.001
Peak HR, bpm	179.23 (±11.37)	181.52 (±7.67)	0.069
Peak VE, L/min	39.42 (±11.80)	39.66 (±10.36)	0.869
RER	1.14 (±0.09)	1.15 (±0.10)	0.253
Peak SBP, mmHg	155.16 (±28.92)	155.95 (±28.86)	0.955
Peak DBP, mmHg	77.43 (±18.28)	80.29 (±18.17)	0.227

Data are the mean ± standard deviation.

AT, Anaerobic Threshold; AT VO_2_, Oxygen Consumption at Anaerobic Threshold; DBP, Diastolic Blood Pressure; FEV1, Forced Expiratory Volume in 1 s; FEV1P, Percentage of predicted Forced Expiratory Volume in 1 s; FVC, Forced Vital Capacity; FVCP, Percentage of Predicted Forced Vital Capacity; HR, Heart Rate; KD, Kawasaki Disease; MVV, Maximal Voluntary Ventilation; Peak VO_2_, Maximum Oxygen uptake measured at Peak Exercise; RER, Respiratory Exchange Threshold; SBP, Systolic Blood Pressure; VE, Minute Ventilation.

Table 3 demonstrates the cardiopulmonary performance of males and females in both the KD and control groups. In the control group, males had higher AT VO_2_ (*p* = 0.005) and peak VO_2_ (*p* = 0.002) than females, which is similar to previous findings.^14^^,^^15^ However, no statistical difference in cardiopulmonary fitness was observed between males and females in the KD group. The authors then further analyzed the cardiopulmonary fitness between the KD and control groups in males and females, respectively. In the male population, the KD group had lower AT VO_2_ (24.74 ± 5.09 mL/min/kg in KD; 27.12 ± 4.63 mL/min/kg in Control, *p* < 0.001) and peak VO_2_ (36.43 ± 7.29 mL/min/kg in KD; 39.94±6.02 mL/min/kg in Control, *p* < 0.001) than the control group. In contrast, in the female population, although the cardiopulmonary fitness of the KD group was worse than that of the control group, the difference did not reach statistical significance.

**Table 3:** Comparisons of baseline characteristic and cardiopulmonary functions between males and females.

	Control			KD			Male			Female		
	Males (*n* = 71)	Females (*n* = 50)	p-value	Males (*n* = 68)	Females (*n* = 49)	p-value	KD (*n* = 68)	Control (*n* = 71)	p-value	KD (*n* = 49)	Control (*n* = 50)	p-value
**Age, years**	9.77 (±1.65)	9.64 (±1.69)	0.662	9.68 (±1.73)	9.59 (±1.58)	0.787	9.68 (±1.73)	9.77 (±1.65)	0.733	9.59 (±1.58)	9.64 (±1.69)	0.884
**BMI, kg/m^2^**	18.79 (±3.29)	18.02 (±3.31)	0.207	19.61 (±4.70)	18.66 (±3.36)	0.201	19.61 (±4.70)	18.79 (±3.29)	0.231	18.66 (±3.36)	18.02 (±3.31)	0.342
**AT VO_2_, mL/kg/min**	27.12 (±4.63)	24.61 (±4.92)	0.005	24.74 (±5.09)	23.80 (±4.92)	0.322	24.74 (±5.09)	27.12 (±4.63)	<0.001	23.80 (±4.92)	24.61 (±4.92)	0.418
**Peak VO_2_, mL/kg/min**	39.94 (±6.02)	36.41 (±6.01)	0.002	36.43 (±7.29)	34.05 (±7.19)	0.082	36.43 (±7.29)	39.94 (±6.02)	<0.001	34.05 (±7.19)	36.41 (±6.01)	0.079
**RER**	1.15 (±0.09)	1.15 (±0.10)	0.851	1.12 (±0.08)	1.15 (±0.10)	0.118	1.12 (±0.08)	1.15 (±0.09)	0.114	1.15 (±0.10)	1.15 (±0.10)	0.978

Data are the mean ± standard deviation.

BMI, Body Mass Index; AT VO_2_, Oxygen consumption at Anaerobic Threshold; KD, Kawasaki Disease; Peak VO_2_, Maximum Oxygen uptake measured at Peak Exercise; RER, Respiratory Exchange Threshold.

## Discussion

This two-center retrospective study investigated the exercise capacity as cardiopulmonary function of prepubertal individuals who had previously experienced KD using a graded treadmill exercise test. The authors observed that patients with a history of KD have lower AT VO_2_ (aerobic capacity) and peak VO_2_ (peak exercise tolerance) than their healthy peers. Furthermore, the authors found that KD more severely affects the cardiopulmonary function of males than that of females. Compared with previous studies on the cardiopulmonary function of patients with KD, which primarily included adolescents,^5^, ^6^, ^7^, ^8^ our study is the first study to focus on prepubertal children.

There are two potential explanations for the difference in CPET findings between the KD and control groups. The first is that it may be related to the long-term effects of KD on the circulatory system. An increasing amount of evidence suggests that there may still be an ongoing intense inflammatory process after the acute stage, resulting in both CA complications and noncoronary complications, such as endothelial dysfunction and myocarditis. Studies have shown endothelial dysfunction in patients with KD that may persist even a decade after the acute stage,^16^, ^17^, ^18^, ^19^, ^20^, ^21^ and these abnormalities have also been reported in patients who have no obvious CA aneurysms detected during the acute stage of KD.^16^^,^^21^^,^^22^ Moreover, studies have found pathological changes in the myocardium, such as myocardial interstitial edema, vasodilatation, and inflammatory cell infiltration during the acute or subacute phase of KD.^23^^,^^24^ Although, most children with KD-associated myocarditis would remain well on follow-up, a few patients still may develop myocardial dysfunction, fibrosis, and myocardial infarction later in life. Furthermore, these manifestations may occur even in patients with no obvious CA aneurysms.^24^, ^25^, ^26^ These studies support our findings that the effects of KD on the cardiovascular system persist years later, which may impact cardiopulmonary function in patients with KD, with or without CA aneurysms.

The second mechanism of decreased oxygen uptake during exercise could be reduced participation in physical activities. Banks et al. found that children with KD have lower weekly Moderate-to-Vigorous Activity (MVPA) levels and lower exercise self-efficacy evaluations than healthy children,^27^ which might negatively affect their cardiopulmonary function later in life. Unfortunately, the authors did not collect data on the participants’ exercise participation and self-rated fitness. Future studies may analyze the association between lower cardiopulmonary function and physical activity levels or self-estimated physical function.

Another important result of our study is that KD affects the exercise capacity of males more than that of females. This can be attributed to the higher incidence rate of CA aneurysms in males; boys with KD outnumbered girls with KD by a ratio of approximately 1.5–1.7:1.^28^ However, there is no convincing explanation for this sex bias until now. As previous studies have reported, children with CA aneurysms are at a higher risk of cardiovascular complications later in life,^29^^,^^30^ which could further reduce their cardiopulmonary function. Unluckily, the authors did not collect the patients’ echocardiographic data, including intraluminal diameters of CA segments and other routinely examined cardiac structures. Therefore, the authors have no idea whether our patients have CA aneurysms and, if so, what the incidence rates were in both sexes. Second, gender stereotypes on sports participation may be another main factor that contributes to the discrepancy in peak VO_2_ between male and female subjects with KD. It has been stated that cultural stereotypes surrounding sports participation, particularly regarding physical strength, prowess, body image, and access to team support, have been suggested to restrict the options available to male individuals. Consequently, this may lead to lower levels of sports-related self-efficacy among males than among females with congenital heart disease.^31^^,^^32^

Although our study results indicated that prepubertal children with KD had lower aerobic capacity and peak exercise tolerance, this does not mean that they cannot engage in exercise normally. The World Health Organization advises that children and adolescents aged 5‒17 years should engage in at least 60 min per day of MVPA, predominantly consisting of aerobic exercises, spread throughout the week.^33^ The ACSM defines vigorous intensity physical activity as > 6 Metabolic Equivalent of Task (MET). In our study, the average peak VO_2_ in the KD group was 35.43 ± 7.31 mL/min/kg, which is equivalent to 10.12 ± 2.09 MET, exceeding the requirements for most vigorous exercises. This indicates that the KD group in our study can safely engage in normal daily activities. However, only 24 % of US youth meet the physical guidelines,^34^ and in Taiwan, where this study was conducted, only 17.8 % of children and youth meet the guidelines.^35^ Therefore, emphasizing the importance of a physically active lifestyle and encouraging children with a history of KD to engage in regular exercise are crucial.

This study has several limitations. First, this was a single-area retrospective study. Although our study included the largest number of cases in this field, a single-area population may lack the external validity required to support widespread changes in clinical practice. Consequently, future studies should recruit participants from multiple locations, even from overseas. Second, data on the patients’ exercise participation level and self-rated fitness are lacking; therefore, the correlation between lower exercise capacity and the physical activity level or self-estimated physical function cannot be analyzed. Third, the authors did not collect patients’ echocardiographic data, including intraluminal diameters of CA segments and other routinely examined cardiac structures. In future studies, the authors will certainly consider incorporating these parameters and other relevant data to provide a more comprehensive understanding.

## Conclusion

Our study revealed that prepubertal individuals with a history of KD had lower aerobic capacity and peak exercise tolerance than the healthy controls. Moreover, the cardiopulmonary function of males is more severely affected by KD than that of females. That being said, prepubertal individuals who once had KD can safely participate in MVPA, although only a small percentage of children meet the physical guidelines, which may negatively impact their cardiopulmonary function later in life. The authors should encourage these patients to engage in exercise consistently and regularly.

## Ethics statement

This study was approved by the Institutional Review Boards of Kaohsiung Veterans General Hospital (number: VGHKS 17-CT11–11, date of approval: 2021/09/27) and Kaohsiung Medical University Hospital (KMUHIRB-E(I)-20,240,166, date of approval: 2024/04/01). All the study adhered to the Helsinki Declaration.

## Authors’ contributions

Yen-Sen Lu: Conceptualization; methodology; formal analysis; writing-original draft; visualization. I-Ching Huang: Conceptualization; supervision; writing-review & editing. Yen-Hsien Wu: Supervision; writing-review & editing. Sheng-Hui Tuan: Validation; supervision; writing-review & editing. Yi-Ching Liu: Supervision; writing-review & editing. Yi-Cheng Wang: Supervision; writing-review & editing. Shih-Hsing Lo: Supervision; writing-review & editing. Jong-Hau Hsu: Conceptualization; supervision; writing-review & editing. Ko-Long Lin: Conceptualization; resources; supervision; writing-review & editing.

## Declaration of competing interest

All authors take responsibility for all aspects of the reliability and freedom from bias of the data presented and their discussed interpretation.
